# What do women want in pharmacy-based HIV prevention services during pregnancy? Developing attributes and levels for a discrete choice experiment in Western Kenya

**DOI:** 10.1186/s12981-025-00752-6

**Published:** 2025-06-04

**Authors:** Melissa Latigo Mugambi, Annabell Dollah, Rosebel Ouda, Nancy Oyugi, Ben O. Odhiambo, Mary M. Marwa, Judith Nyakina, John Kinuthia, Bryan J. Weiner, Grace John-Stewart, Ruanne Vanessa Barnabas, Brett Hauber

**Affiliations:** 1 Department of Global Health, University of Washington, Seattle, USA; 2 Kenyatta National Hospital, Nairobi, Kenya; 3 UW-Kenya, Nairobi, Kenya; 4 Safe Water AIDS Project, Kisumu, Kenya; 5 Department of Research and Programs, Kenyatta National Hospital, Nairobi, Kenya; 6 Department of Medicine, University of Washington, Seattle, USA; 7 Department of Epidemiology, University of Washington, Seattle, USA; 8 Department of Pediatrics, University of Washington, Seattle, USA; 9 Division of Infectious Diseases, Massachusetts General Hospital and Harvard Medical School, Boston, USA; 10 The Comparative Health Outcomes, Policy and Economics (CHOICE) Institute, Department of Pharmacy, University of Washington, Seattle, USA; 11 Pfizer, Inc, New York, NY USA

## Abstract

**Background:**

The delivery of HIV prevention services (e.g., HIV testing, pre-exposure prophylaxis (PrEP) initiation and refills, and STI testing) in community pharmacies could address clinic barriers faced by pregnant women such as extended travel and wait times. We conducted a qualitative study in Western Kenya to select and prioritize attributes and levels for a discrete choice experiment (DCE) to design pharmacy-based HIV prevention services for pregnant women.

**Methods:**

We began by identifying a comprehensive list of attributes and levels relevant to women considering HIV prevention during pregnancy. This list was informed by recommended HIV prevention interventions for pregnant women, our objective to design services for pharmacy settings, and attributes identified in the literature as important for other populations when choosing HIV and pharmacy-based services. From March to November 2022, we recruited participants using stratified purposeful sampling and collected qualitative data through seven focus group discussions with women, four with health providers, and eight individual interviews with technical experts. Interviews were audio-recorded, translated, transcribed, and summarized in debrief reports. We conducted debriefing meetings and analyzed these reports to identify and refine the essential attributes that would influence decisions to access HIV prevention services from a pharmacy during pregnancy.

**Results:**

We initially identified twelve potential attributes that were evaluated during the focus groups. Five attributes were eliminated based on ranking exercises with women and health providers. Additional attributes suggested during the focus groups were ranked low by participants or not mentioned frequently enough and, therefore, not included. We finalized and refined levels for each attribute using insights from the literature, participant feedback, and design considerations. The study identified seven attributes: service location, type of HIV test, STI testing availability, partner HIV testing availability, PrEP availability, service access methods (e.g., appointment versus walk-ins), and service fee.

**Conclusions:**

This study was the first step in data collection toward defining attributes and levels for a DCE survey and successfully identified seven preliminary attributes and levels. Pre-testing and pilot testing activities further explored the feasibility and understanding of the attributes and levels.

**Supplementary Information:**

The online version contains supplementary material available at 10.1186/s12981-025-00752-6.

## Introduction

In Kenya, adolescent girls and young women increasingly purchase or prefer purchasing various sexual and reproductive health products from pharmacies, such as HIV self-tests, oral contraceptives, and, more recently, pre-exposure prophylaxis (PrEP), citing pharmacy accessibility and convenience [1–4]. Although there are ongoing efforts to improve access to HIV prevention products in healthcare settings, including PrEP for pregnant women – a critical at-risk population - pharmacies might expand the reach of these services and provide additional options for women during the pregnancy journey [5]. We need to explore whether pharmacies can be beneficial in improving access to HIV prevention services among pregnant women and creating value in the pregnancy care continuum.

Currently, the National Ministry of Health in Kenya is exploring the potential for retail pharmacies to dispense PrEP as part of its strategic plan to improve the uptake of HIV prevention services [6]. Two pharmacy models have been evaluated in Kenya: one involves employing trained pharmacy providers to identify eligible clients, discuss HIV risk, perform HIV tests, and dispense PrEP with guidance from remote providers; the other model incorporates nurse navigators to support these services [7–9]. While PrEP is consistently supplied through Ministry of Health channels in public settings, the financing strategy for private-sector delivery is still being explored. This includes considerations for clients’ out-of-pocket costs and potential public-private partnerships [6]. 

Qualitative research is essential in providing insights into what attributes women value and prioritize in seeking HIV prevention services [10–12]. Program planning and implementation stakeholders can also help identify plausible service attributes to improve uptake and impact [8]. In circumstances where there is no pre-defined question about the service to develop, the research might focus on understanding what women value and what they would like to see in HIV prevention services and then deriving attributes based on these concepts and tailoring the service to meet their unique needs and preferences – a bottom-up approach [10]. However, where there are published guidelines on the proposed service or examples of similar services, we can use these resources as a starting point to identify existing attributes and qualitatively evaluate their importance and impact on women’s preferences [12]. We can then use these attributes to design discrete choice experiments (DCEs), simulate the services that women would choose based on the attributes, and quantify the importance of these attributes in service design [13]. 

Our overall goal in this study was to develop a DCE to inform the design recommendations of pharmacy-based HIV prevention services for women during pregnancy. Our qualitative research objective was to determine which attributes are relevant to women’s potential use of such services so that we can include the necessary and relevant attributes in the DCE.

## Methods

### Study team

We conducted the study as a multidisciplinary team with expertise in maternal health, HIV prevention, pharmacy-based services, and DCE methods. Our team included physician-scientists (MLM, JK, RVB, GJS), nurse-scientists (JN, BOO), economists (RVB, BH), epidemiologists (GJS, MMM), and social research scientists (AD, RO, NO, BJW). Collectively, we have extensive experience working with the study population in Western Kenya.

### Study setting

This study was conducted in Kenya, focusing on three counties in the western region: Homa Bay, Kisumu, and Siaya. As of 2019, these counties have a combined population of approximately 3.2 million, representing about 6.9% of Kenya’s total population. Homa Bay and Siaya are predominantly rural, while Kisumu is the largest urban center in the region, with more developed infrastructure and greater access to healthcare services. In terms of socioeconomic status, these regions experience higher poverty rates than Kenya’s more urbanized areas [14, 15]. This is particularly true in the rural communities of Homa Bay and Siaya, where health infrastructure is more limited. Kisumu, being more urbanized, has somewhat better healthcare access and infrastructure [16]. 

According to the 2022 Kenya Demographic and Health Survey, the percentage of 15 to 19-year-old women who had ever been pregnant was higher in Homa Bay (23.2%) and Siaya (20.9%) than in Kisumu (11.1%) and Kenya on average (14.9%). As of 2023, the HIV prevalence among females aged 15–49 years was also higher in these counties. In Siaya, it was 12.7%; in Homa Bay, it was 13.8%; and in Kisumu, it was 14.9% compared to the national average of 4.5% [17]. 

A majority of women aged 15–49 in Kisumu (68%) and Siaya (79%) reported receiving antenatal care services from a nurse, midwife, or clinical officer. In Homa Bay, this percentage was slightly lower at 49%, with 47% reporting services from a doctor. Most women in these counties reported delivering their babies in public sector clinics: 59% in Homa Bay, 77% in Kisumu, and 71% in Siaya. In Homa Bay, 28% of women reported delivery in private-sector clinics [17]. 

Access to health facilities varied by county, with just over half of the women in Kisumu (58%) and Homa Bay (52%) reporting travel times of less than 30 min, compared to 66% in Siaya reporting travel times of 30 min to 2 h [17]. 

### Study population and sampling

We conducted focus group discussions (FGDs) with a stratified purposeful sample of women who had participated in the PrEP Implementation for Mothers in Antenatal Care (PrIMA) study in Homa Bay and Siaya, as well as a pharmacy PrEP pilot in Kisumu [9, 18]. We recontacted potentially eligible participants via phone and invited them to participate in the FGDs. Focus groups were stratified by age (adolescent girls, young women, and older women) and recruitment source: women who had participated in pharmacy PrEP studies in Kisumu and PrIMA studies in Homa Bay and Siaya. Due to recruitment challenges, one adolescent group was recruited through snowball sampling in Kisumu.

Women were eligible to participate if they self-reported as HIV-negative, were aged 15–44, had been pregnant or intended to become pregnant within the next five years, and were willing to join the study. Women under 18 were eligible only if they had experienced pregnancy, given their status as emancipated minors. We purposefully stratified focus groups with health providers, including community health workers and nurses who provided care at PrIMA study health facilities. Participants were selected for inclusion with guidance from the in-charges at each health facility. Each focus group comprised 5–10 participants. We conducted individual interviews with technical experts purposefully selected for their knowledge of maternal health, HIV prevention, and pharmacy-delivered services. All participants were reimbursed for their time after each interview.

### Approach to the development of attributes and levels

We used a systematic approach to develop attributes and levels (similar to that) described by Helter et al., consisting of raw data collection, data reduction, removing inappropriate attributes and wording and refinement [11]. 

#### Step 1: Raw data collection

We identified a comprehensive list of attributes (and potential levels where possible) relevant to women when considering HIV prevention services during pregnancy. This was informed by the types of HIV prevention interventions recommended for women during pregnancy, our research objective to design services for a new location—pharmacies versus antenatal care clinics—and attributes that have been shown to be important in other populations when choosing HIV and pharmacy-based services [19–22]. 

Overall, we identified 12 preliminary attributes. We were particularly interested in how to design pharmacy-based HIV prevention services; therefore, we included an attribute describing the location (pharmacy vs. antenatal care clinic) where women would access the services. There were five HIV prevention attributes reflecting approaches recommended by global and national guidelines to reduce a woman’s risk of acquiring HIV during pregnancy. These approaches included testing the woman for HIV, testing the woman’s partner for HIV, testing for sexually transmitted infections (STIs), offering PrEP initiation and PrEP refills [7, 19]. Given the regulatory barriers that limit pharmacy providers from conducting rapid HIV diagnostic testing that is required to initiate PrEP, an alternative approach might focus on expanding access to PrEP refill services; therefore, we initially included the availability of pharmacy-based refills as an attribute [23]. HIV self-testing is increasingly available in pharmacies, offering options like blood-based finger prick tests and an oral swab tests therefore, we assessed whether these types of HIV tests might influence women’s choice of service [4, 24, 25]. 

We also identified other attributes from the literature that have been particularly important in influencing the uptake of PrEP in pharmacies in pilot studies, including health provider availability (and type of provider), private room availability, service fee, how long one waits for the service, when the pharmacy is open (operation hours) and follow-up - the approach used to follow up with a client after receiving a service [1, 20, 26, 27]. This initial list of attributes served as the basis for discussion and prioritization during subsequent focus groups with women and healthcare providers, as well as interviews with technical experts.

#### Step 2: Data reduction and removal of inappropriate attributes

We initially conducted seven FGDs with women to refine the list of attributes and then four FGDs with health providers to validate the findings from the discussions with women. Before each discussion, participants completed a consent form and a brief survey. Two trained social research scientists fluent in English, Kiswahili, and Dholuo facilitated the FGDs following a semi-structured interview guide, supported by two notetakers. One facilitator and one notetaker were assigned to each group. The discussions were primarily conducted in English but were translated into Kiswahili and Dholuo as needed.

During these discussions, participants identified barriers and facilitators to clinic- and pharmacy-based services, rated the importance of the attributes identified in step 1 (“very important,” “somewhat important,” or “not at all important”), and explained the reasons behind their preferences. Participants also identified preliminary attribute levels and rank-ordered the attributes individually and as a group to identify the attributes that were most important to them. We iteratively updated the interview guide to include topics requiring more probing or to evaluate refined attributes and levels. All interviews were audio-recorded, translated (as needed), transcribed, and summarized in debrief reports.

After each interview, we (MLM, AD, RO, NO, JN, MMM, BO, and BH) analyzed the debrief reports and identified and refined essential attributes that would inform decisions about accessing HIV prevention services from a pharmacy during pregnancy. We systematically reviewed the data to determine which attributes to retain, modify, or exclude. We used several approaches to narrow down the attributes to 6–8, a standard threshold in DCE studies, to reduce the risk of cognitive fatigue among participants when selecting potential services. These approaches included removing attributes ranked low in importance by participants, considered infeasible for real-world implementation, or likely to dominate choice tasks [11]. We used a point-based system to rank attributes. In each FGD with women, participants collectively ranked the service attributes, with the highest-ranked attribute receiving 1 point, the second-highest 2 points, and so on. Attributes with the lowest total scores were considered the highest ranked.

#### Step 3: Wording and refinement

After the FGDs, we developed a preliminary set of attributes and levels. A trained social research scientist then interviewed technical experts to get their perspectives on designing a pharmacy-based service for delivering HIV prevention services during pregnancy and to ask for their feedback on the feasibility of the preliminary attributes and levels. The interviews also informed further refinement of these attributes and levels. We then developed an initial DCE questionnaire and conducted individual cognitive think-aloud interviews with women to pre-test the questionnaire and ensure that the language used for the selected attributes and levels was meaningful and easily understood. Findings from the pre-testing will be presented in a separate manuscript.

### Ethical approval

The study was approved by the Kenyatta National Hospital – University of Nairobi Ethical Research Committee, the National Commission for Science, Technology, and Innovation (NACOSTI), and the University of Washington Institutional Review Board. All participants provided written informed consent before participating in the study.

## Results

### Participant characteristics

From March to May 2022, we conducted seven focus group discussions with 52 women of reproductive age. The median age of the women was 24 (IQR: 19, 32). Twenty-four women (46%) were married or living with a partner, 25 (48%) were unemployed, 38 (73%) had previously been pregnant, and 37 (71%) visited a pharmacy at least every two or three months (Table 1).

**Table 1:** Characteristics of women enrolled in the study

					Total		
						(*n* = 52)	
Age (y)			Median (interquartile range)		24.0		(19.0, 32.0)
Relationship status			Married or living with a partner		24		(46%)
			Single and never married		20		(38%)
			Divorced/separated/widowed		1		(2%)
			Has a partner but not cohabiting		7		(13%)
Education status			Primary school		7		(13%)
			Secondary school		14		(27%)
			University/college		12		(23%)
			Polytechnic		4		(8%)
			Not currently enrolled		15		(29%)
Employment status			Salaried		9		(17%)
			Regular hourly work		4		(8%)
			Irregular hourly work		10		(19%)
			Unemployed		25		(48%)
Pharmacy visits in the past year			Every month		16		(31%)
			Every 2 or 3 months		21		(40%)
			1 or 2 times a year		13		(25%)
			Never		2		(4%)
Travel time to regularly visited pharmacy			Less than 15 min		35		(67%)
			15–29 min		8		(15%)
			30–59 min		8		(15%)
			≥ 60 min		1		(2%)
Prior pregnancy			Yes		38		(73%)
			No		14		(27%)
Number of pregnancies			Median (interquartile range)		2.5		(1, 4)

In May 2022, we conducted four focus group discussions with 39 health providers. Health providers had a median age of 38 (IQR: 32.0, 45.0). Nineteen (49%) were nurses, and 20 (51%) were community health workers. Twenty-eight providers (72%) were female. In terms of educational attainment, 4 (10%) had completed primary school, 11 (28%) had completed high school, 10 (26%) had completed vocational training, and 14 (36%) had completed university (bachelor’s degree) (Table 2).

**Table 2:** Characteristics of health providers enrolled in the study

					Total		
						(*n* = 39)	
County			Siaya		19		(49%)
			Homa Bay		20		(51%)
Gender			Male		11		(28%)
			Female		28		(72%)
Age (years)			Median (interquartile range)		38		(32, 45)
Highest level of education attained			Primary education		4		(10%)
			Secondary/high education		11		(28%)
			Trade/technical/vocational training		10		(26%)
			Bachelor’s degree		14		(36%)
Current job title			Nurse		19		(49%)
			Community health worker		20		(51%)
Duration in current position (years)			Median (interquartile range)		8		(3, 12)

From October to November 2022, we conducted individual interviews with eight technical experts. The technical experts comprised 6 women and 2 men aged 32 to 54. The participants had training in nursing, pharmacy, medicine, and social sciences, as well as experience providing clinical care to pregnant women and implementing HIV prevention in pharmacies and public health facilities gained through research and programmatic work.

The findings from the interviews with women, healthcare providers, and technical experts were instrumental in identifying a preliminary list of attributes and levels for the DCE. The focus group discussions with women primarily informed the list of attributes and levels. Feedback from healthcare providers served as a validation step and did not result in significant changes to the identified attributes and levels. However, input from the technical expert interviews led to adjustments, as discussed below.

### HIV prevention attributes

We refined and reduced the attributes to include four HIV prevention interventions: HIV testing, STI testing, partner HIV testing, and PrEP. Most participants indicated that having these interventions available in pharmacy-based HIV prevention programs for pregnant women was important.

*“There should be a one-stop pharmacy as in where when a pregnant mother comes*,* she gets like everything where she is seated. I come*,* you give me…you offer me antenatal services*,* if you offer me prevention services for HIV*,* then if I turn HIV positive after testing*,* I am able to receive my ARV drugs within the same same room I entered. The same room should also be able to provide me with STI screening and if I would need PrEP*,* I would also be able to come out with it from the same same room.”*

*(FGD 8*,* Nurse*,* Homa Bay*,* P9)*.

### HIV testing

Participants perceived offering HIV testing services as important for maternal health and enhancing efforts to prevent mother-to-child transmission.

*“I think that it is very important to go to the pharmacy for a HIV test once you get to learn that you are pregnant because I might be living far from the hospital…So*,* the most important thing is to go to the pharmacy early enough so that one can learn how to take care of that pregnancy.”*

*(FGD 7*,* Woman*,* Siaya*,* P7)*.

There were mixed preferences for blood-based and oral swab-based self-tests.

Some participants preferred blood-based tests due to their accuracy:

*“I would prefer blood testing*,* because it is very fast and more accurate.”*

*(FGD 4*,* Woman*,* Siaya*,* P3)*.

In contrast, some participants preferred oral swabs because they were not painful (like finger prick tests) and could be used in the privacy of one’s home.

*" …doing it at home by myself preferably yeah*,* especially the ones that have come orally…At home I feel like I’m alone. I can simply do it and see my results. Privacy is also at home compared to the pharmacy*,* and maybe I might even be afraid of the syringe.”*

*(FGD 6*,* Woman*,* Kisumu*,* P6)*.

Because HIV testing is increasingly available in pharmacies and participants had mixed preferences, we initially defined an HIV testing attribute with two levels: *oral swab self-tests* and *blood-based self-tests*. Technical experts agreed with the initial test types but also suggested including provider-assisted testing for the blood-based tests.

*“If it’s going to be possible to have this trained person within the pharmacy also have the knowledge or have training in HIV testing*,* then I will not mind also having the rapid diagnostic tests within the pharmacies.”*

*(Technical Expert Interview 1*,* Male*,* 32 years)*.

Therefore, we expanded the levels for this attribute to include *blood-based self-tests*,* blood-based provider-assisted tests*, and *oral swab self-tests*.

### STI testing

STI testing was valued for protecting both maternal and child health:

*“There are a lot of STIs like HPV*,* Gonorrhea*,* I feel like they’re very important so that it can protect you and the child as well as a pregnant woman and you know through these STIs they have*,* which can transmit HIV. So I feel like the screening is also important because it can help in prevention…"*.

*(FGD 6*,* Woman*,* Kisumu*,* P1)*.

Regarding how they wanted STI testing to be provided, preferences were mixed. Some preferred to collect their samples at home, while others thought it would be better in a pharmacy setting. Regarding the sample method, many participants expressed discomfort with vaginal swab tests, preferring less invasive procedures like urine-based options. In some cases, blood-based tests were also preferred over vaginal swabs, as participants perceived them to be more private. Given that STI testing is not typically available in pharmacies, we concluded that the DCE would need to assess the marginal utility of service availability—that is, whether women would be interested in receiving this service in a pharmacy setting. Therefore, STI testing had two levels describing the *availability* or *unavailability* of services.

### Partner HIV testing

Providing partner HIV testing at the pharmacy was viewed as important because it allowed both partners to know their status, build trust, and overcome the fear associated with visiting clinics.

*“I feel that it is good for us to go together to the chemist (pharmacy) because most of these men are usually afraid of going to public hospitals and they also fear queueing. But you know at the chemist*,* we will just go together and we get attended to fast enough.”*

*(FGD 7*,* Woman*,* Siaya*,* P9)*.

Similar to HIV testing, there were mixed preferences for blood-based versus oral swab tests. Some participants preferred the blood-based (rapid test) due to its accuracy and familiarity while others preferred the oral swab due to its convenience. Partner HIV testing is not available in pharmacies; therefore, we created two levels indicating whether partner HIV testing would be *available* or *not available*.

### PrEP initiation and refills

Finally, access to PrEP was perceived as important because of the benefits of preventing HIV.

*“It is important to get PrEP from the pharmacy because I could be pregnant and I am not yet ready to go to the hospital. Now PrEP would be of help to me to prevent myself from HIV as I wait for my time to go for my antenatal care.”*

*(FGD 7*,* Woman*,* Siaya*,* P3)*.

Regarding how they wanted PrEP to be delivered, many participants preferred injections over oral pills because they did not like taking pills, and injections would reduce the frequency of visits for refills. One participant suggested that choice would be important to cater to different women’s preferences. Given that PrEP was not available in pharmacies at the time, the DCE was designed to explore the marginal utility of PrEP availability and PrEP refills. To reduce the number of HIV prevention attributes, we initially combined PrEP and PrEP refills into one attribute called PrEP availability, which included three levels: *PrEP initiation and refills*, *PrEP refills only*, and *no PrEP available*.

Experts’ opinions on PrEP service delivery at pharmacies were mixed. While some supported pharmacy-only refills for capacity-constrained settings (e.g., where no private room is available) or for highly mobile populations who might initiate PrEP elsewhere, others advocated for full-service pharmacies to minimize client confusion and costs. Flexibility in options and the ability for women to choose were also emphasized.

*“I think there were studies that were designed like that and that was the idea. Like you can start pharmacy in a public health facility*,* but you come for refills in pharmacies*,* which is okay because…that was…testing a system: is it even possible to do PrEP in pharmacies? But once you’ve discovered that it’s possible to do it in pharmacy*,* then just provide all of it and women can choose or people can choose where to start and where to continue.”*

*(Technical Expert Interview 3*,* Female*,* 44 years)*.

We decided that PrEP services should have two levels—*fully available (PrEP initiation and refills)* or *unavailable*—because if a pharmacy is being introduced as another delivery point comparable to antenatal care clinics, it should not be limited to offering refills only. Moreover, if the providers are trained, implementing full-scale PrEP services at pharmacies is feasible [7]. 

In one focus group discussion with nurses, the issue of offering post-exposure prophylaxis (PEP) for HIV was perceived as important. In two focus group discussions, both nurses and community health workers highlighted the importance of providing condoms, while in one focus group with women, female condoms were also mentioned. PEP was not mentioned frequently enough in the interviews to warrant its inclusion as an attribute. Rather than include female condoms as an attribute, we included a survey question to evaluate whether it might be a potential product that women would like their regular pharmacy to offer.

### Service attributes

Overall, participants suggested that pharmacies had the potential to offer more convenient and accessible care options. However, some participants raised concerns about privacy and disclosure of test results and questioned whether pharmacy providers were adequately trained to provide such services.

*“Let’s say for example you have your partner and you don’t want people to know.* *If you go* *to the pharmacy the two of you, and maybe the pharmacy attendant knows you. What if he goes and says it somewhere or tell someone that knows* *you?”*

*(FGD 1*,* Woman*,* Kisumu*,* P3)*.

Among the service features, having a health provider was ranked as the most important, followed by a private room, service fee, and operation hours. A health provider and private room primarily addressed women’s concerns about privacy, confidentiality, and the need for competent providers in pharmacy settings.

“*It’s very important (to have a health provider) because like I said, some of the people are not qualified to work so they may not know what services to provide like the trained nurse.”*

*(FGD 5*,* Woman*,* Kisumu*,* P2)*.

*“I put private rooms is number one because one of the major things that came out clearly in all the services we were talking about was stigma and confidentiality. So I think private rooms would take care of that.”*

*(FGD 4*,* Woman*,* Siaya*,* P1)*.

We concluded that women would likely choose a service if they knew a qualified health provider was present, even if the service lacked other desired attributes. As a result, we decided to eliminate the presence or absence of a qualified health provider as an attribute. Instead, we asked participants to assume that a qualified health provider would be available irrespective of the location as part of introducing the discrete choice experiment.

Privacy was an important factor for women and likely would significantly influence their choice of service location. Rather than including privacy as a separate attribute, we redefined it as a level within the location attribute to assess whether women derive additional utility from accessing care at a pharmacy without a private room compared to an antenatal care clinic, as not all pharmacies offer private rooms. We additionally included follow-up survey questions to assess women’s perception of privacy among different service locations.

Most participants considered a service fee ranging from 50 to 250 Kenyan Shillings (KSH) affordable but indicated that a free option would be preferred. Some participants were willing to pay up to KSH 500.

“*It’s not important for me to pay [for PrEP] because the government is giving out. I want it for free.”*

*(FGD 3*,* Woman*,* Homa Bay*,* P6)*.

*“Let us be realistic. At the community level*,* we usually observe the trend of how the community pharmacies access customers…the buying and the uptake*,* usually the uptake is high. And both girls and women*,* whatever services they do obtain from the pharmacy*,* they are never for free. They pay. There are many a times when some of them charge even 200 shillings for some reproductive health services and those women go for them.”*

*(FGD 10*,* Community Health Volunteer*,* Homa Bay*,* P8)*.

“*Depending on the price range, yes. Because if you sell it, let’s say at 1500 shillings, not everybody can afford that 1500 shillings. But let’s say you sell it at 250 or 500, I believe someone can really struggle so much to get that 500 shillings and get the PrEP to…help.”*

*(FGD 6*,* Woman*,* Kisumu*,* P1)*.

Technical experts agreed that KSH 300 was affordable and that KSH 500 might be somewhat pricey based on the economy at the time. However, in one instance, an expert indicated that women might be willing to pay as much as KSH 800. We therefore included three service fee levels: free, KSH 300, and KSH 500.

Most women preferred flexible facility operating hours, including nights and weekends. Results from ongoing pilot studies also suggested that providers preferred this option, as HIV prevention visits take longer [20]. Therefore, we initially proposed three levels for the operating hours attribute reflecting service provision during work hours only, or also after work hours, and after work hours and weekends.

Technical experts, however, found the initial timeframes unrealistic for antenatal care clinics. To avoid a restricted analysis, we changed the opening hours attribute to reflect the fraction of time services are available (how one accesses the services) with three levels: walk-ins, coming in at a set day or time each week, or making an appointment [28]. 

We excluded the attributes “wait time” and “follow-up” because women ranked them low, indicating that pharmacy wait times were usually short and unlikely to affect their decision to use the service and expressed privacy concerns around follow-up or felt that it should be treated on a case-by-case basis. The final attributes included location, how one accesses the services, and service fee.

Overall, we identified seven attributes for inclusion in the DCE through our qualitative approach to attribute and level development (Table 3). The next step, as part of a separate study, involved pre-testing and piloting the DCE survey to confirm that we selected attributes relevant to choosing an HIV prevention service, evaluate the clarity and comprehension of the choice tasks, and examine how the attributes and levels influenced participants’ preferences [5]. 

**Table 3:** Preliminary attributes and levels to define service alternatives that were identified from the study

Attribute	Levels
1. The location where the services are provided	1. You go to the antenatal care clinic 2. You go to a community pharmacy with a private room 3. You go to a community pharmacy without a private room
2. The type of HIV test you use	1. You test yourself with an oral swab test 2. You test yourself with a blood-based finger prick test 3. A provider tests you with a blood-based finger prick test
3. Whether partner HIV testing is available	1. Partner HIV testing is not available 2. Partner HIV testing is available
4. Whether STI testing is available	1. STI testing is not available 2. STI testing is available
5. Whether PrEP is available	1. PrEP is not available 2. PrEP is available
6. How you get the services	1. You make an appointment 2. You walk in anytime when the location is open 3. You walk in on specific days and times of the week
7. How much you pay for the services	1. You do not pay anything for the services 2. You pay 300 KSH for the services 3. You pay 500 KSH for the services

## Discussion

Our study aimed to design a DCE survey to inform the development of pharmacy-based HIV prevention programs in Kenya. By evaluating women’s preferences for HIV prevention services during pregnancy, we aimed to identify pharmacy service offerings that could make them a viable alternative to clinics. Prior research shows that priority populations exhibit heterogeneous preferences for how HIV prevention services should be delivered and that the ability to choose preferred services enhances the likelihood of uptake [29]. As a first step in designing the DCE survey, we took a systematic qualitative approach to capture what attributes are most relevant to women of reproductive age in choosing an HIV prevention service during pregnancy. The qualitative approach was critical for refining the experiment and ensuring we did not exclude key elements needed to design a valid preference measurement instrument.

In this study, the attribute “location” was directly related to the core objective; therefore, it was important to confirm whether it might influence the selection of an HIV prevention service. Overall, participants had mixed preferences for receiving services in a pharmacy versus an antenatal care clinic during pregnancy. Additionally, participants were open to receiving services from a pharmacy, especially if certain needs were met, such as privacy and the availability of trained, competent providers.

To our knowledge, no other studies have designed a discrete choice experiment to evaluate women’s preferences for receiving HIV prevention services during pregnancy. However, in a similar study in Malawi focused on evaluating preferences for PrEP services among female sex workers, participants ranked delivery location as a priority attribute, emphasizing elements such as convenience and accessibility [29]. In a qualitative evaluation to better understand barriers to pregnancy test use in Western Kenya, participants had mixed preferences for the location to obtain pregnancy testing services, with some women appreciating the convenience and accessibility of services from pharmacies. In contrast, other women perceived that antenatal care clinics provided better access to skilled personnel [30]. Nonetheless, our findings confirm that key design features, including the availability of a private room or access to a trained provider proposed by decision-makers in piloting pharmacy-based models in Kenya, also matter to women who might seek HIV prevention services from pharmacies during pregnancy [8]. 

While it is unclear how many pharmacies have private rooms in this study setting, qualitative evidence suggests variability in the type of pharmacy premises, ranging from smaller one-room stalls with client interactions occurring over the counter to larger pharmacies with a private space or room [31]. In this study, privacy was an important factor in women’s willingness to use pharmacy services. However, given that not all pharmacies have private rooms, it was critical to assess whether, all else being equal, women would choose a pharmacy without a private room over a clinic – that is - whether there might be additional value in providing such services in pharmacies lacking private rooms compared to those with private rooms.

Interestingly, most women in this study did not rank wait time as an important attribute compared to others. In similar discrete choice experiments, wait time has typically been perceived as important and included in the final list of attributes [21, 29]. While including “wait time” as a separate attribute would have provided more insight into location preferences, we limited our attributes to all HIV prevention intervention attributes and those prioritized by women, health providers, and technical experts. We included additional questions outside of the DCE to understand further why women might choose a particular location.

We acknowledge that we recruited participants from three counties in Western Kenya (Kisumu, Homa Bay, and Siaya), and most of these participants had previously participated in PrEP implementation studies in maternal and child health clinics or pharmacy settings, so their perspectives might not necessarily generalize to other settings [32]. However, we used a systematic approach incorporating insights from the literature, followed by qualitative interviews and ranking exercises with several stakeholder groups, including adolescent girls, young women, older women, health providers, and technical experts. The attributes developed represent an initial step in identifying key drivers of choice among women considering HIV prevention services. To ensure their relevance and feasibility, these attributes were further evaluated through cognitive interviews and a pilot study before finalizing the design of the DCE survey [32]. Altogether, these steps ensured that the final instrument was valid, and when combined into profiles, the attributes were robust enough to allow women to make meaningful choices and provide the insights needed to inform the design of pharmacy-based HIV prevention services for this population.

## Conclusions

Our study represents the first step in data collection toward defining attributes and levels for a DCE survey and successfully identified seven preliminary attributes and levels. We used a systematic approach, including the preliminary identification of attributes from the literature, followed by focus groups and ranking exercises with diverse stakeholders. Overall, participants expressed heterogeneous preferences for having HIV prevention services available at the pharmacy. Our findings informed a follow-up DCE survey to quantify and assess women’s preferences for the type of HIV prevention services in pharmacies and how they should be delivered.

## Electronic supplementary material

Below is the link to the electronic supplementary material.

Supplementary Material 1

Supplementary Material 2

## Data Availability

The datasets used and/or analysed during the current study are available from the corresponding author on reasonable request.

## Abbreviations

ANC: Antenatal Care
DCE: Discrete Choice Experiment
FGD: Focus Group Discussion
HIV: Human Immunodeficiency Virus
KSH: Kenyan Shilling
PEP: Post-Exposure Prophylaxis
PrEP: Pre-Exposure Prophylaxis
