# Supplementary material for: What do women want in pharmacy-based HIV prevention services during pregnancy? Developing attributes and levels for a discrete choice experiment in Western Kenya
Source: AIDS Res Ther. 2025 Jun 4;22:58. doi: 10.1186/s12981-025-00752-6 (PMC12139256; doi:10.1186/s12981-025-00752-6)
Supplement: Supplementary file 1 — Supplementary Material 1 [file 12981_2025_752_MOESM1_ESM.docx]

INTERVIEW GUIDE FOR SEMI-STRUCTURED INTERVIEWS WITH DECISION-MAKERS

**Preferences for delivering HIV prevention interventions in community pharmacy settings**

I am interviewing you today to validate some of the findings we have heard from women of reproductive age in Kisumu, Homa Bay, and Siaya counties about their thoughts on obtaining HIV prevention services in pharmacy settings. Based on those interviews, we identified several HIV prevention interventions and service characteristics for pregnant women, and we would like your feedback.

Before I get started, I would like to learn more about you and your role.

1. To start off with, what is your current job title?
2. How long have you been in your current position?
3. What is the highest degree or level of school you have completed?
4. How many years you have been practicing as a [insert role]?
5. If you don’t mind me asking, how old are you?

Consider a program that will enable women to receive HIV prevention services during pregnancy in a community pharmacy setting instead of going to the antenatal care clinic. These services include HIV testing, STI screening, partner testing for HIV, and PrEP and **will be provided by a trained individual**.

1. What do you think of this idea?
2. Why do you think some pregnant women might choose to obtain HIV prevention services in community pharmacies?
3. What might prevent pregnant women from seeking such services in community pharmacies?
4. What problems or difficulties are pregnant women likely to encounter when trying to obtain HIV prevention services in community pharmacies?
5. **What would a successful pharmacy-based HIV prevention program for pregnant women look like? What would make it a viable alternative to the traditional antenatal care clinic-based programs? For example, do we need the majority of women to choose such a program? Do we simply need another option that’s just as good as existing ANC services? Do we need another option for some types of women?**
6. What HIV prevention services makes sense to provide for pregnant women in community pharmacies?

We have compiled a list of possible HIV prevention services to offer in community pharmacies. We plan to present potential users with questions asking them to choose among alternative services. Each service will be described by various characteristics, which we will discuss in this interview. When given a choice, we assume potential users will select a service based on the characteristics that are important to them. We will use the data to determine which HIV prevention services to include and how to deliver HIV prevention services for pregnant women in a pharmacy setting. Here is an example of a question a potential user might see:

**Question 1**

***In service A, you are tested for HIV using an oral swab test, you can be tested for STIs, your partner can be tested for HIV, you cannot start PrEP but when you run out of PrEP pills you get a refills, you receive the service in a community pharmacy with a private room, you can access the service from Monday to Sunday between 7am and 10pm and you pay 300 KES.***

*Alternatively, in service B* ***you are tested for HIV using a blood-based finger prick test, you can be tested for STIs, your partner can be tested for HIV, you can start PrEP and when you run out of PrEP pills you get a refills, you receive the service in the antenatal care clinic, you can access the service from Monday to Friday between 8am and 5pm and the services are free of charge – you do not pay anything.***

***Which one would you choose?***

|  | **Service A** | **Service B** |
| --- | --- | --- |
| **The type of HIV test you use** | An oral swab self-test | A blood-based finger prick provider assisted test |
| **Whether you can be tested for sexually transmitted diseases** | Yes, you can be tested for sexually transmitted diseases | Yes, you can be tested for sexually transmitted diseases |
| **Whether your partner can be tested for HIV** | **Yes, your partner can be tested for HIV** | **Yes, your partner can be tested for HIV** |
| **The type of PrEP services that you receive** | You cannot start PrEP, but when you run out of PrEP pills, you can get a refill | You can start PrEP pills and get a refill (more pills) when you run out. |
| **The location where the services are provided** | A community pharmacy with a private room | An antenatal care clinic |
| **The day and time that you are able to access the services** | **Monday to Sunday, 7am – 10pm** | **Monday to Friday, 8am – 5pm** |
| **How much you pay for the services** | You pay 300 KES for the services | The services are free of charge - you do not pay anything |
| Which service would you choose? | | |
|  Service A |  Service B |  Neither |

We would like to get your thoughts on these service components.

**HIV testing**

Firstly, I would like to talk about HIV testing.

1. Would having HIV testing as a service in a community pharmacy make a difference for pregnant women? What difference does it make? Why?
2. We include the following options for HIV testing: 1) an oral swab self-test, which is saliva-based, and 2) a blood-based finger prick self-test and 3) a provider-assisted rapid diagnostic test (also blood-based) . What do you think of the HIV test type options we have chosen? Is the distinction among these types of tests important? Is one type of test likely to be more important from a policy or programmatic standpoint?
3. Do you think we should include any other types of HIV tests?
4. Do you think the types of HIV tests offered by pharmacies will change in the future?

**STI screening**

1. Would having STI screening as a service in a community pharmacy make a difference for pregnant women? What difference does it make? Why?
2. To what extent are STI screening services available in community pharmacies?
3. Will there be women interested in this type of service in the community? What types of women are likely to be interested?

**Partner HIV testing**

1. Would having partner HIV testing as a service in a community pharmacy make a difference for pregnant women? What difference does it make? Why?
2. To what extent are partner HIV testing services available in community pharmacies?
3. Will there be women interested in this type of service in the community? What types of women are likely to be interested?

**PrEP services**

1. Would having PrEP as a service in a community pharmacy make a difference for pregnant women? What difference does it make? Why?
2. There are three potential options that women can select in the survey based on the location:

1) women can start PrEP pills and get a refill (more pills) when they run out

2) women cannot start PrEP, but when they run out of PrEP pills, they can get a refill, and

3) women cannot get started on PrEP or get a refill.

What do you think of the PrEP service options we have chosen?

1. Are these PrEP service options important? Do they make sense?
2. Is it important to make a distinction among whether one can start PrEP and get a refill VS. one can only get a refill VS. one cannot start PrEP or get a refill at a particular location? Is it necessary to understand women’s preferences for a service with PrEP initiation and refills versus another only offering PrEP refills?
3. In practice, can we have a scenario whereby pharmacies only offer PrEP refills but not PrEP initiation services?
4. Do you think we should include any other PrEP service options?
5. How are PrEP service options likely to change in the future?

**Other**

1. Are there any other HIV prevention services that we should have included here?
2. Are there any concerns about the HIV prevention services we have included?

Next, I would like to discuss some of the service characteristics that women expressed as important to them during preliminary focus group discussions (show table with list of characteristics).

**Facility location**

1. For the location of the services, women can choose among 1) an antenatal care clinic, 2) a community pharmacy with a private room, and 3) a community pharmacy without a private room. In practice, can we have a scenario where HIV prevention services can be offered in pharmacies with a private room and in pharmacies **without** private rooms?
2. Is it necessary to understand women’s preferences for a service provided by a community pharmacy **without** a private room versus for a service provided by an antenatal care clinic? Why?
3. Is there anything you would change about the facility locations we have included?

**Operating hours**

1. We have included the following day and time options to receive services: 1) Monday to Friday, 8 am – 5 pm, 2) Monday to Friday, 7 am – 10 pm, 3) Monday to Sunday, 7 am – 10 pm. Are the day and time options we have provided likely to be realistic in community pharmacy settings? What day and time options are realistic in antenatal care clinics?
2. Is it helpful to consider walk-in options – people can walk in and receive a service anytime? Or is it likely that services will only be available on certain days of the week or during the day in pharmacy settings? Do you think it will be possible for women to schedule appointments for specific days/times during the week?
3. Is there anything you would change about this particular attribute to reflect the extent to which services are available to women?

**Service fee**

1. Do you think the service fee will likely influence a pregnant woman's decision to obtain HIV prevention services from the pharmacy vs. the antenatal care clinic? Why?
2. Do you think it makes sense to be charged an overall service fee vs. a fee for each type of HIV prevention service?
3. We have included three options for the service fee: women are not charged anything - that is - the service is free; 2) the service costs 300 KES, and 3) the service costs 500 KES. Are these amounts realistic?
4. What is the maximum amount women can realistically be charged for HIV prevention services in a pharmacy, assuming a flat fee? What is the minimum amount they can realistically be charged in the pharmacy? Is it safe to assume that services in the antenatal care clinic will be free of charge? Do you foresee the amount people pay for services changing in the future, either in the pharmacy or antenatal care clinic? Why?
5. Is there anything you would change about the service fees we have included?

**Closing questions**

1. Are there any concerns or comments about the service characteristics we have included?
2. Is there anything you would change about the service characteristics and options we've selected?
3. Are there any other factors that might influence a pregnant woman's decision of whether to participate in a pharmacy-based HIV prevention program?

**Table 1: List of potential service characteristics**

| **SERVICE** | **OPTION** |
| --- | --- |
| 1. **The type of HIV test you use** | There are two types of HIV tests you might use:   - - An oral swab test SELF-TEST, which is saliva-based, requires you to swab the upper and lower gums of your mouth.   - A blood-based finger prick SELF-TEST - requires **you to prick your finger and obtain a blood sample for the test**.   - A provider assisted rapid diagnostic test |
| 1. **Whether you can be tested for sexually transmitted diseases such as Syphilis, Gonorrhea, and Chlamydia** | There are two options available:   - - Yes, you can be tested for sexually transmitted diseases.   - No, you cannot be tested for sexually transmitted diseases. |
| 1. **Whether you partner can be tested for HIV** | There are two options available:   - - Yes, your partner can be tested for HIV   - No, your partner cannot be tested for HIV |
| 1. **The type of PrEP services that you receive.** | There are three options for PrEP services:   - - You can start PrEP pills and get a refill (more pills) when you run out.   - You cannot start PrEP, but when you run out of PrEP pills, you can get a refill.   - You cannot get started on PrEP or get a refill. |
| 1. **The location where the services are provided** | There are three locations where you might choose to obtain HIV prevention services during pregnancy:   - An antenatal care clinic. A woman might regularly visit an antenatal care clinic when she is pregnant, so health providers can check that the woman and her baby are healthy. - A community pharmacy with a private room - there is a dedicated space where private discussions can take place. - A community pharmacy without a private room - there is no dedicated space where private discussions can take place. Interactions with the pharmacy provider will take place over the counter. |
| 1. **The day and time that you are able to access the services**. | There are three options to when you can access services:   - - **Monday to Friday, 8am – 5pm**   - **Monday to Friday, 7am – 10pm**   - **Monday to Sunday, 7am – 10pm** |
| 1. **How much you pay for the services** | By this, we mean the standard fee that you pay for all HIV prevention services. There are three options:   - - The services are free of charge - you do not pay anything   - You pay 300 KES for the services   - You pay 500 KES for the services |
