# Supplementary material for: What do women want in pharmacy-based HIV prevention services during pregnancy? Developing attributes and levels for a discrete choice experiment in Western Kenya
Source: AIDS Res Ther. 2025 Jun 4;22:58. doi: 10.1186/s12981-025-00752-6 (PMC12139256; doi:10.1186/s12981-025-00752-6)
Supplement: Supplementary file 2 — Supplementary Material 2 [file 12981_2025_752_MOESM2_ESM.docx]

**INTERVIEW GUIDE FOR FOCUS GROUP DISCUSSIONS WITH WOMEN AND HEALTH PROVIDERS**

**Preferences for delivering HIV prevention interventions in community pharmacy settings**

The objective of this section is to identify and prioritize factors that should be considered in designing HIV prevention services for pregnant women in community pharmacy settings. Specifically, we want to understand the types of service features that clients would value and would influence their decision in opting whether to obtain HIV prevention services from a community pharmacy. Findings from this section will also help us to identify additional features that we have not considered.

**TOPIC 1: Socio-demographics**

[For health providers and decision-makers. To completed individually before the health provider FGD]

1. To start off with, could you tell me how many years you have been practicing as a [insert role]?
2. What is your current job title?
3. How long have been in your current position?
4. What is the highest degree or level of school you have completed? (If currently enrolled, highest degree received.)
5. If you don’t mind me asking, how old are you?
6. Have you recently completed any training or participated in any research projects on HIV prevention or antenatal care/reproductive health?

[For women – to be completed individually before the FGD]

1. What is your relationship status?
2. Please state your age in years
3. What is your current grade level in school?
4. If not currently enrolled in school or if attending short courses, what is your highest level of education completed?
5. What is your employment status?
6. How much money do you usually earn in a month?
7. Have you ever been pregnant?
8. How many times have you been pregnant?
   1. Number of pregnancies ending in live birth
9. When did your last pregnancy end?
10. How long does it usually take you to get to the antenatal care clinic that is closest to your home?
11. What mode of transportation do you use?
12. How long does it typically take you to get to the community pharmacy or chemist that is closest to your home?
13. What mode of transportation do you use to commute to the pharmacy?
14. How often do you visit a community pharmacy during the year?

**TOPIC 2: Barriers and facilitators to uptake of HIV prevention services and interventions in pharmacy settings**

1. What kinds of problems or difficulties have you ever come across when trying to obtain services in community pharmacies? Please use the following services as probes to assess barriers. Please make sure to note if a barrier is specific to a service **[Note: A helpful approach for those who have not accessed the services in question would be to ask, would you receive (insert service e.g., STI screening) in a pharmacy? What would be the barriers to such a service in the pharmacy?)**].
   1. Pregnancy testing
   2. Counseling women based on pregnancy test results
   3. Provider assisted HIV testing or HIV self-testing
   4. Partner testing for HIV
   5. PrEP initiation
   6. PrEP refills
   7. STI screening/testing
   8. Any other reproductive or HIV prevention services?
2. What kinds of problems or difficulties have you ever come across when trying to obtain these services in health facilities? Please use the following services as probes to assess barriers. Please make sure to note if a barrier specific to a service.
   1. Pregnancy testing
   2. Counseling women based on pregnancy test results
   3. Provider assisted HIV testing or HIV self-testing
   4. Partner testing for HIV
   5. PrEP initiation
   6. PrEP refills
   7. STI screening/testing
   8. Any other reproductive or HIV prevention services?
3. What advantages do you see to obtaining these services in community pharmacies? [**Note:** a) if the issue of **confidentiality** comes up in neighborhood pharmacies, confirm if they would be willing to go to a pharmacy further away from their home area versus a health facility; b) if the issue of **privacy** comes up, confirm whether privacy is better in a pharmacy versus a health facility and why; c) if the issue of **stock outs** come up, confirm whether the problem is worse in a pharmacy versus a health facility; d) if the issue of **provider attitudes** come up, confirm who is more approachable pharmacy providers or health facility providers; e) if the issue of **counseling** comes up, ask them what they mean by counseling].
4. What advantages do you see to obtaining these services in health facilities?

**TOPIC 3: Preferences for HIV prevention services and interventions**

Consider a program that will enable women to receive HIV prevention interventions during pregnancy in a community pharmacy setting instead of going to the antenatal care clinic.

1. What HIV prevention services or interventions do you think would be important to make available in a pharmacy-based HIV prevention program for pregnant women? [Note: please provide examples as needed to facilitate discussion with the participants.].
   1. How important is providing HIV testing services (very important, somewhat important, not at all important)? Why? How do you want HIV testing services to be provided? Why? (*For HIV testing services, this could be a blood-based test in which the client’s finger is pricked, an oral saliva-based test done at the pharmacy with guidance, or an oral saliva-based test that the woman takes home to do on her own. To help guide the discussion, the interviewer can ask questions like – how about a blood-based test which you conduct at the pharmacy? How about an oral saliva-based test that you conduct at the pharmacy? )*
   2. How important is providing STI screening services (very important, somewhat important, not at all important)? Why? How do you want STI screening services to be provided? Why? *(For STI screening, the facilitator can discuss the use of point-of-care diagnostic devices such that samples – vaginal swabs or blood through a finger prick are collected in the pharmacy* ***or self-collected at home in the case of vaginal swabs; also might be helpful to discuss urine samples****)*.
   3. How important is providing partner testing services (very important, somewhat important, not at all important)? Why? How do you want partner testing services to be provided? Why? *(Partner testing involves receiving counseling and taking back several HIV self-tests to conduct with a partner.)*
   4. How important is providing PrEP initiation services (very important, somewhat important, not at all important)? Why? How do you want PrEP initiation services to be provided? Why? *(PrEP initiation will involve doing an HIV test, risk screening/counseling, and based on this receiving PrEP pills that are taken once daily. Other options in the future might include a pill that’s taken once a month or an injection that is administered every 2 months)*
   5. How important is providing PrEP refill services (very important, somewhat important, not at all important)? Why? How do you want PrEP refill services to be provided? Why? *(PrEP refill services will involve going back to the pharmacy for more pills or another injection when the pills are finished or when another injection is due)*

**TOPIC 4: Preferences for delivery of HIV prevention services and interventions in pharmacy settings**

Consider a program that will enable women to receive HIV prevention interventions during pregnancy in a community pharmacy setting instead of going to the antenatal care clinic.

1. Do you have any thoughts on what service features (or characteristics) would be important to you in deciding whether to use a pharmacy-based HIV prevention program for pregnant women? **[Note: please provide examples as needed to facilitate discussion with the participants].**
   1. How important is the service fee to you (very important, somewhat important, not at all important)? Why? What is the most you (pregnant women) are willing to pay for such a service?
   2. How important are the operation hours of the pharmacy when the services are provided (very important, somewhat important, not at all important)? Do you have any preferences for what days the services should be provided? Do you have any preference for the hours during which the services should be provided? How about early morning e.g. 6am – 8am? How about in the evening e.g., after 5pm? Why?
   3. How important is the amount of time you (or pregnant women) spend at the pharmacy (very important, somewhat important, not at all important)? Do you have any preference for how long you (or pregnant women) are willing to spend at the pharmacy? How about 10 minutes? How about 30 minutes? How about 1 hour? Why?
   4. How important is having a health provider e.g., a nurse or community health worker connected at the pharmacy to discuss any concerns or questions pregnant women might have (very important, somewhat important, not at all important)? Do you have any preferences for how the health provider should interact with the women? How about in-person in a private room at the pharmacy? How about via phone? How about via text message? Why? **[Note: might be helpful to assess which type of provider is more approachable – a nurse, pharmacy provider or HTS counselor etc.]**
   5. How important is the ability to communicate with providers about any questions or concerns you might have when you are not at the pharmacy (i.e., remotely)? (very important, somewhat important, not at all important)? Why? How does it help you? Do you have any preferences for how you would like to communicate with providers? How about via text message/SMS? How about calling via phone? Why?
   6. How important is having a private room within the pharmacy to discuss any health-related issues (very important, somewhat important, not at all important)? Why? Do you have any preferences for where health-related discussions with providers should occur? How about via phone?
   7. Are there any other features that are important to you and might influence your decision of whether to participate (or whether pregnant women should participate) in a pharmacy-based HIV prevention program? How about the payment options available for the service?

**TOPIC 5: Ranking exercise**

We are now going to conduct an exercise to rank the features that you consider to be important or somewhat important in designing a pharmacy-based HIV prevention service for pregnant women. You will rank the features individually and then as a group (if a focus group discussion), by assigning a numerical rank to each feature. For example, the most important feature will be ranked no. 1 and the next most important feature will be ranked no. 2 and so on.

*Following completion of the ranking exercise the interviewer will lead a discussion to understand the rationale behind the rankings.*

Sample questions: Why were these 4 features ranked highly? Why was this feature ranked as the most important? Why was this feature ranked as the least important?
